# Supplementary material for: CARD9 negatively regulates NLRP3-induced IL-1β production on Salmonella infection of macrophages
Source: Nat Commun. 2016 Sep 27;7:12874. doi: 10.1038/ncomms12874 (PMC5052644; doi:10.1038/ncomms12874)
Supplement: Supplementary Figures and Supplementary Table — Supplementary Figures 1-22 and Supplementary Table 1 [file ncomms12874-s1.pdf]

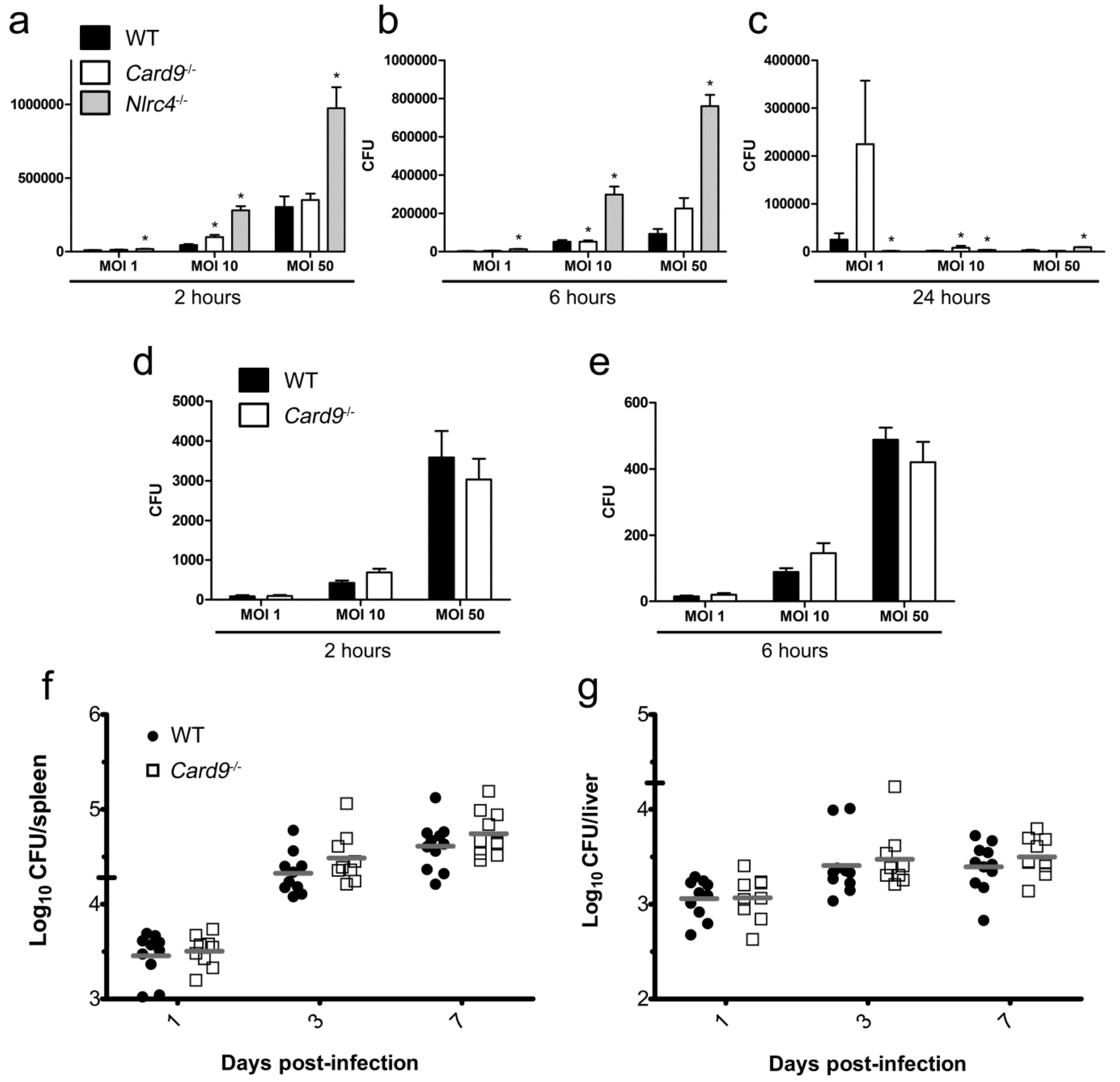

Supplementary Figure 1: CARD9 does not influence bacteria counts *in vitro* and *in vivo.* (a-c) intracellular bacteria counts of WT, *Nlrc4*$^{-/-}$ and *Card9*$^{-/-}$ BMDMs after infection with *S*. Typhimurium SL1344 at MOIs 1, 10 and 50 for 2 (a), 6 (b) and 24 (c) hours. (g-h) Bacteria burden in the spleen (g) and liver (h) after C57BL/6 WT and *Card9*$^{-/-}$ infection with *S*. Typhimurium M525P (4x10^3 CFU). * $p<0.05$ in comparison to WT (one-way ANOVA with Tukey's multiple comparisons test). (a-e) Data from two independent experiments (mean and s.e.m.). (g-h) Data from two independent experiments using from four to six mice per genotype, plus two negative controls per genotype. Mice were from 8 to 16 weeks old, both male and female.

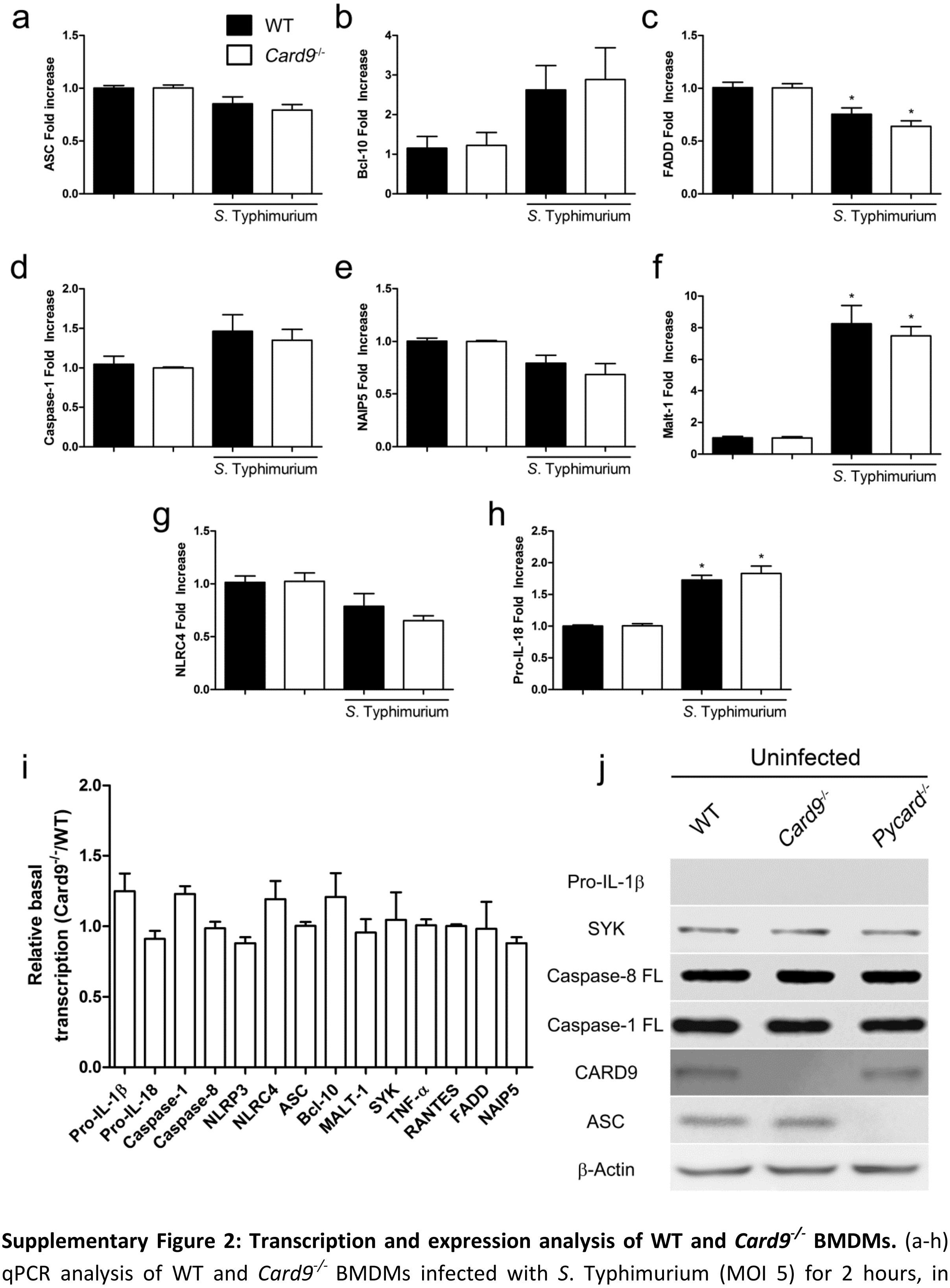

Supplementary Figure 2: Transcription and expression analysis of WT and *Card9$^{-/-}$* BMDMs. (a-h) qPCR analysis of WT and *Card9$^{-/-}$* BMDMs infected with *S*. Typhimurium (MOI 5) for 2 hours, in relation to their respective uninfected controls. (a) ASC, (b) Bcl-10, (c) FADD, (d) Caspase-1, (e) NAIP5 , (f) Malt-1, (g) NLRC4, (h) pro-IL-18. (i) Basal transcriptional levels in uninfected *Card9$^{-/-}$* BMDMs in relation to uninfected WT cells. (j) Basal expression of pro-IL-1β, SYK, Caspase-8 Full Length, Caspase-1 Full Length, CARD9, ASC, β-actin in WT, *Card9$^{-/-}$* and *Pycard$^{-/-}$* BMDMs cell lysates. * $p<0.05$ in

comparison to uninfected control (one-way ANOVA with Tukey's multiple comparisons test). (a-i) Data from three independent experiments (mean and s.e.m.). (j) Image is representative of three independent experiments.

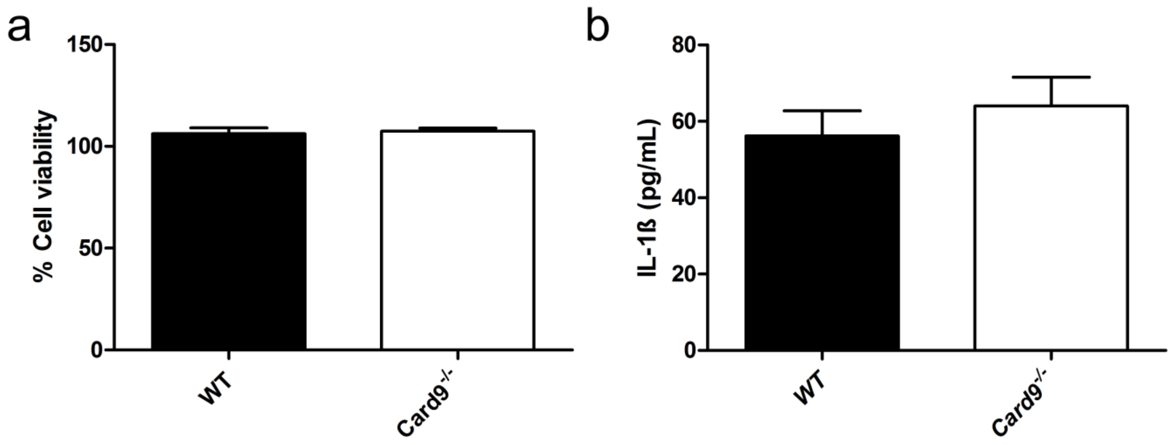

Supplementary Figure 3: CARD9 does not control IL-1β produced via AIM2. (a) Cellular viability and (b) IL-1β from LPS-primed BMDMs after transfection with poly(dA:dT) for 4 hours. Data from three independent experiments (mean and s.e.m.).

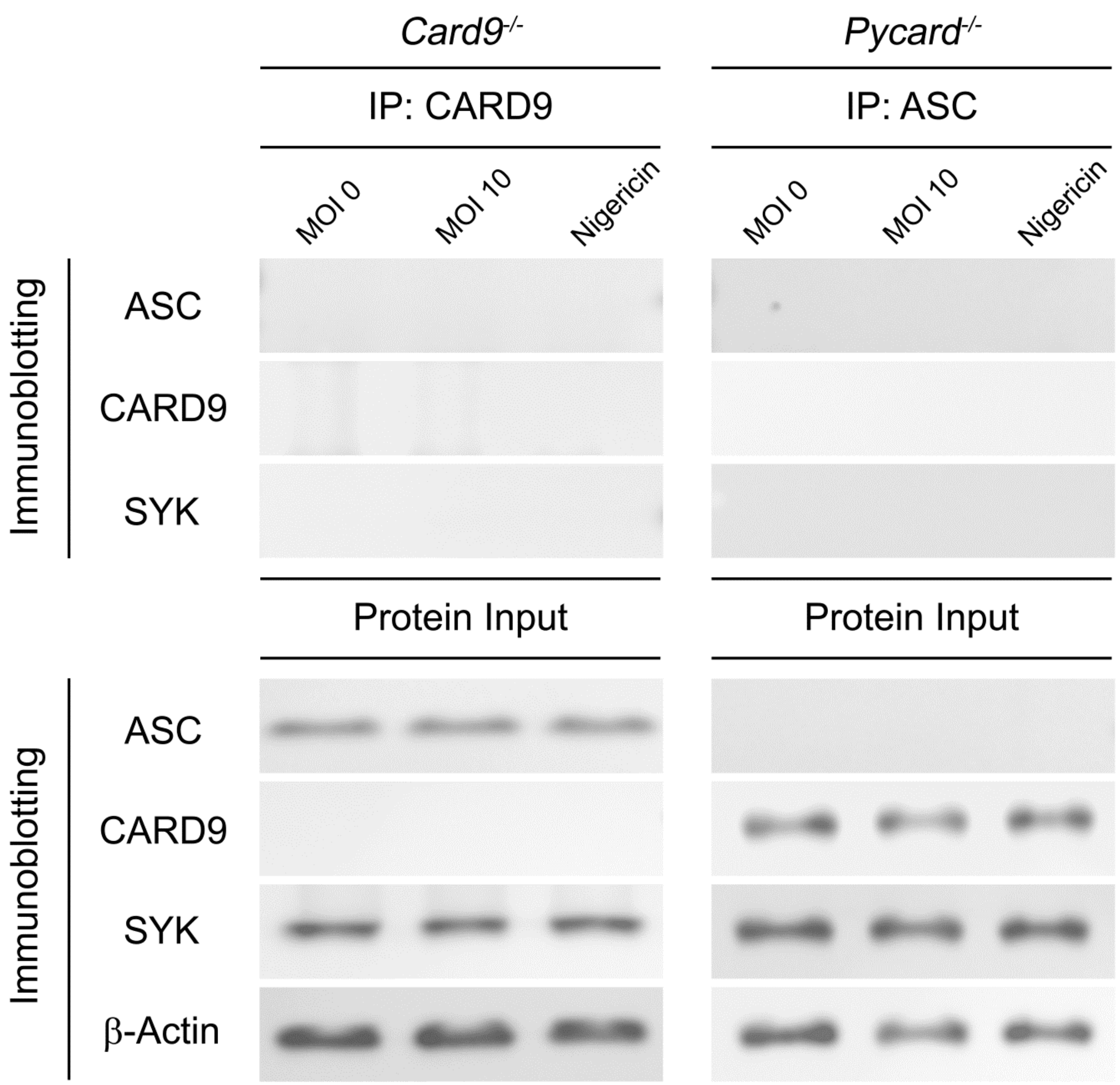

Supplementary Figure 4: CARD9 and ASC co-IPs isotype controls. *Card9*$^{-/-}$ and *Pycard*$^{-/-}$ BMDMs were primed with LPS (200 ng/mL) for 3 hours and incubated with *S*. Typhimurium (MOI 10, 30 minutes) or with Nigericin (10 µM, 30 minutes). IPs were then performed as indicated using anti-CARD9 (for *Card9*$^{-/-}$ BMDMs) or anti-ASC (for *Pycard*$^{-/-}$ BMDMs), followed by immunoblotting. No IPs or co-IPs are observed. Images are representative of three independent experiments.

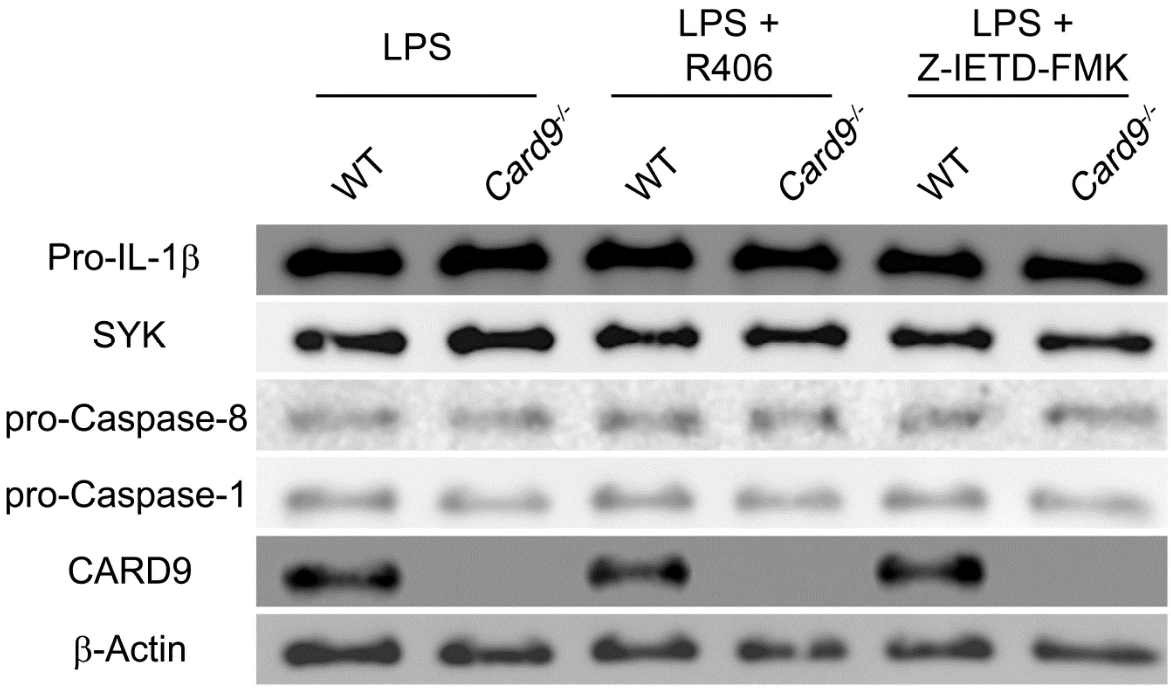

Supplementary Figure 5: LPS-priming by itself or use of Z-IETD-FMK and R406 do not cause different expression in WT and *Card9*$^{-/-}$ BMDMs. Expression of pro-IL-1β, SYK, pro-caspase-8, pro-caspase-1, CARD9, β-actin in of cell lysates from WT and *Card9*$^{-/-}$ BMDMs incubated for 3 hours with LPS (200 ng/mL) or 3 hours with LPS with and additional hour of incubation with Z-IETD-FMK (10 µM) or R406 (1 µM). Figure is representative of three independent experiments.

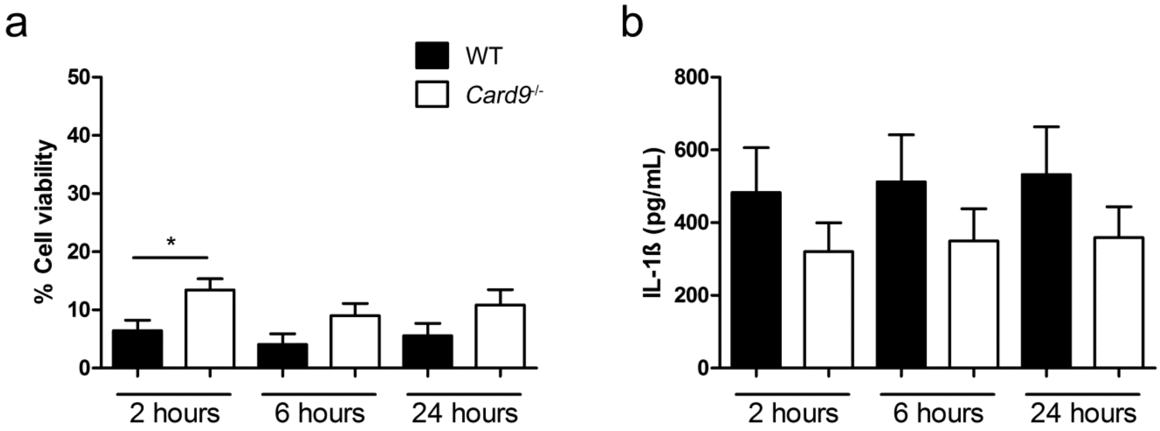

Supplementary Figure 6: CARD9 plays no role in controlling IL-1β production in BMDCs. (a) cellular viability (as measured by LDH release) and (b) IL-1β secretion (as measured by ELISA), of WT and *Card9*$^{-/-}$ BMDCs after infection with *S*. Typhimurium SL1344 at MOI 10 for 2, 6 and 24 hours. (a-b) Data from three independent experiments (mean and s.e.m.).

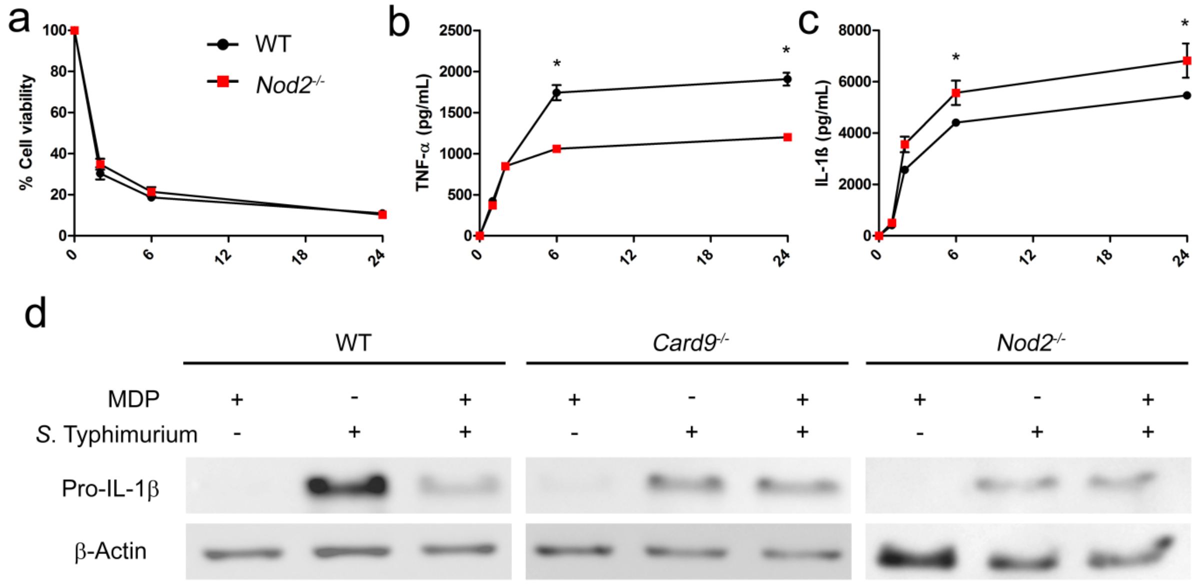

Supplementary Figure 7: NOD2 up- and down-regulates the expression of different cytokines during *S*. Typhimurium infection. (a) Cellular viability (as measured by LDH release) (b) IL-1β secretion (as measured by ELISA) and (c) TNF-α (as measured by ELISA) of WT and *Nod2*$^{-/-}$ BMDMs after infection with *S*. Typhimurium SL1344 at MOI 10 for 2, 6 and 24 hours. (d) Pro-IL-1β expression in WT, *Nod2*$^{-/-}$ and *Card9*$^{-/-}$ BMDMs infected with S. Typhimurium (MOI 10) with or without MDP co-stimulation (10 µg/mL) (a-c) Data from two independent experiments (mean and s.e.m.). (d) Figure is representative of three independent experiments. * $p<0.05$ (two-way ANOVA) with Bonferroni post-test.

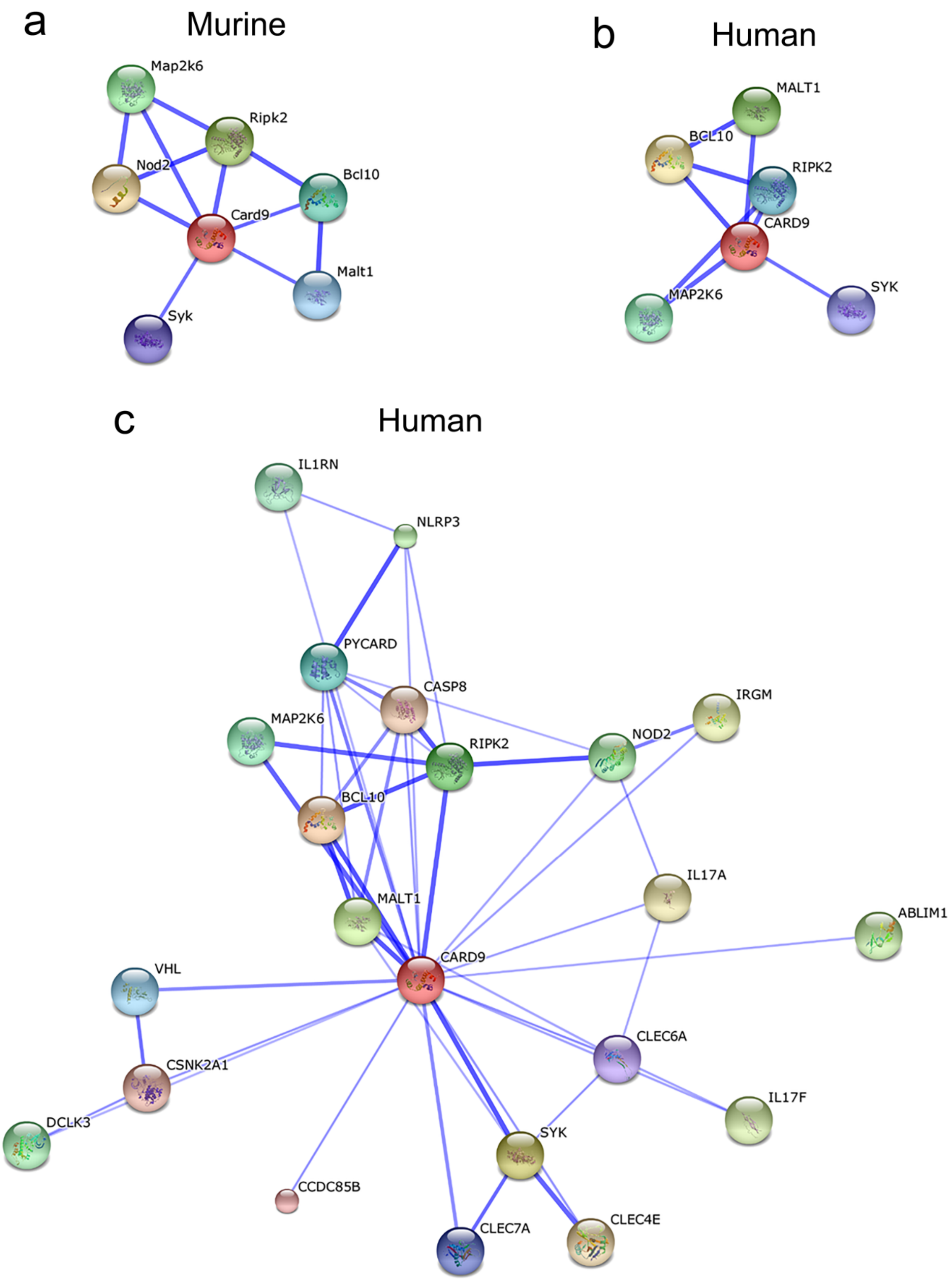

Supplementary Figure 8: CARD9 interfaces with multiple inflammatory and death-related signalling pathways. Functional association network analysis of the primary CARD9 interactome was performed using STRINGv10. High confidence interaction partners (score > 0.7) for murine (a) and human (b) CARD9. Reducing the stringency of the inclusion criteria to include medium confidence interaction partners (score > 0.4) confirms the expansive signalling network in which CARD9 is involved supporting its assignment as a critical inflammatory signalling node (c).

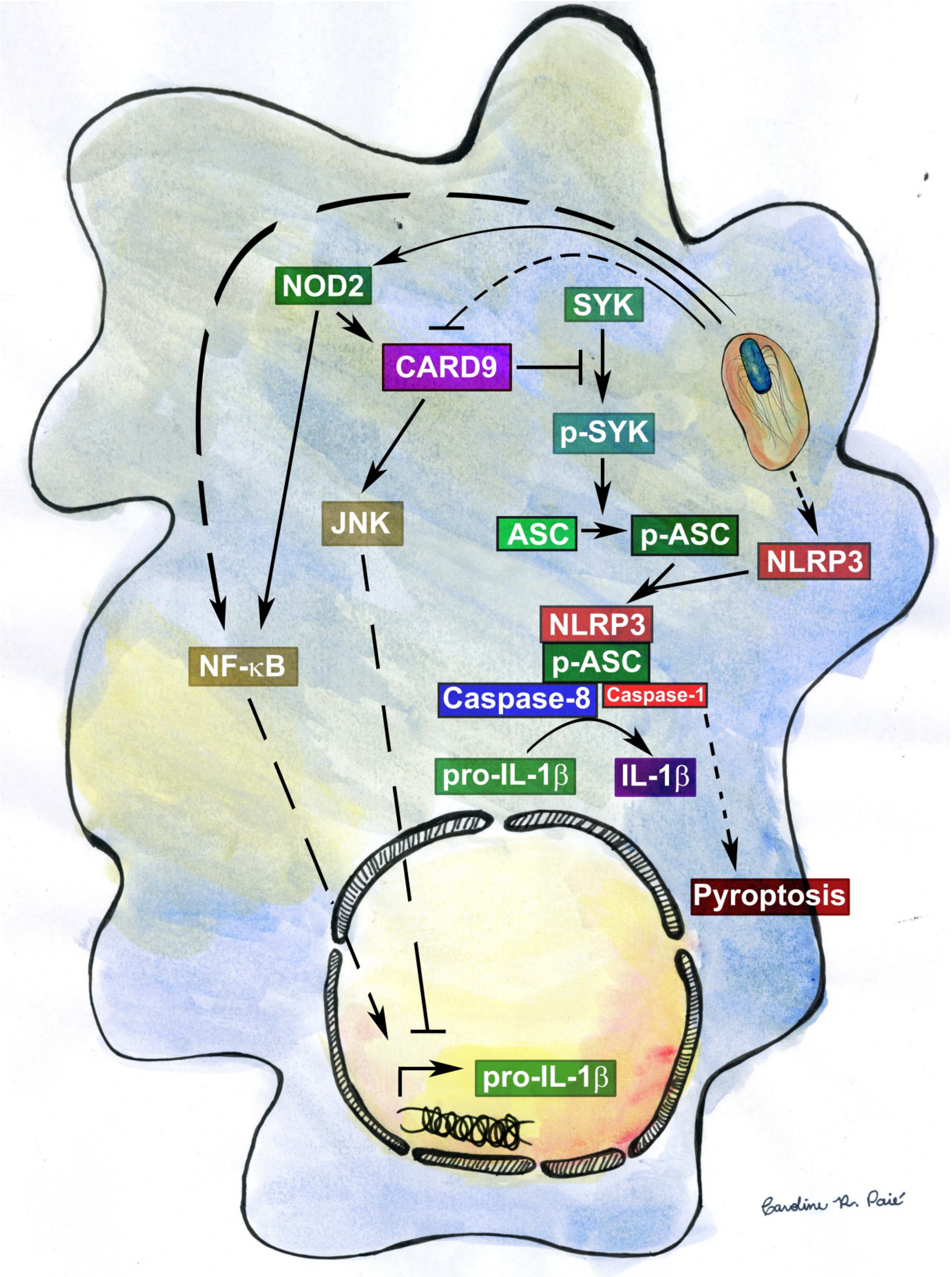

Supplementary Figure 9: Simplified model for CARD9 regulation of IL-1β production. Infection by *S.* Typhimurium triggers the assembly of the NLRP3 inflammasome in the cytoplasm of BMDMs with subsequent processing of pro-IL-1β to mature IL-1β. CARD9 negatively regulates inflammasome activity at two levels: suppressing pro-IL-1β expression and reducing caspase-8-dependent IL-1β processing.

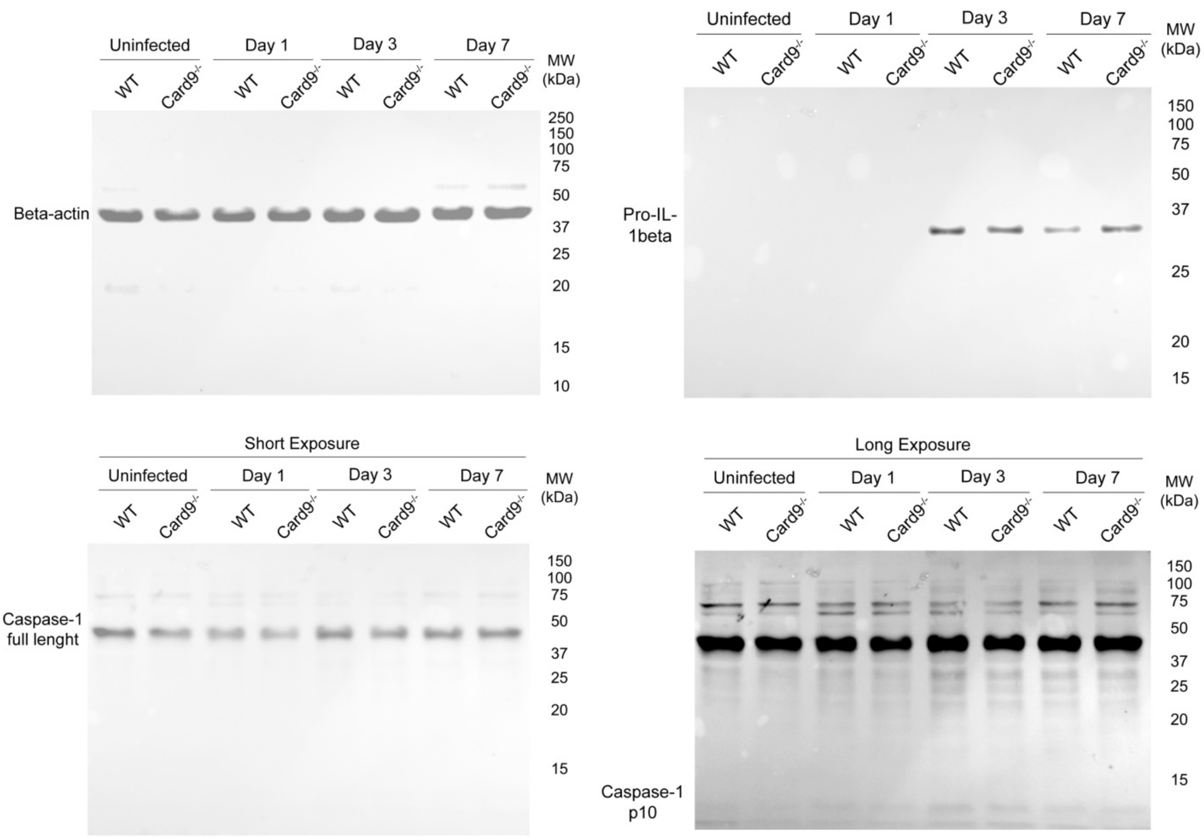

Supplementary Figure 10: Uncropped immunoblots presented in Figure 1-p. Membranes were probed using the antibody indicated (β-Actin, Pro-IL-1β and Caspase-1).

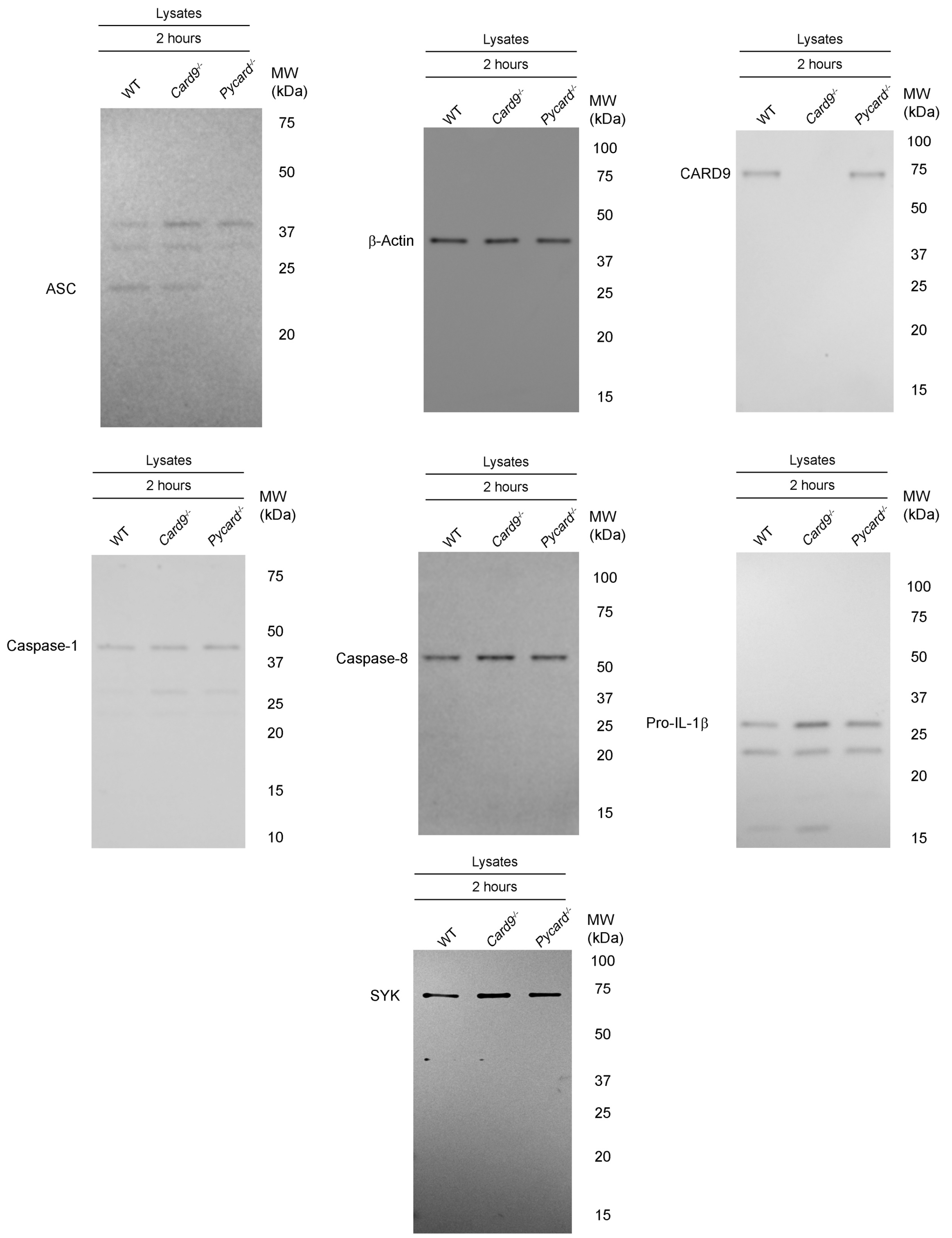

Supplementary Figure 11: Uncropped immunoblots presented in Figure 2-g, Supernatants. Membranes were probed using the antibody indicated (ASC, β-Actin, CARD9, Caspase-1, Caspase-8, Pro-IL-1β and SYK).

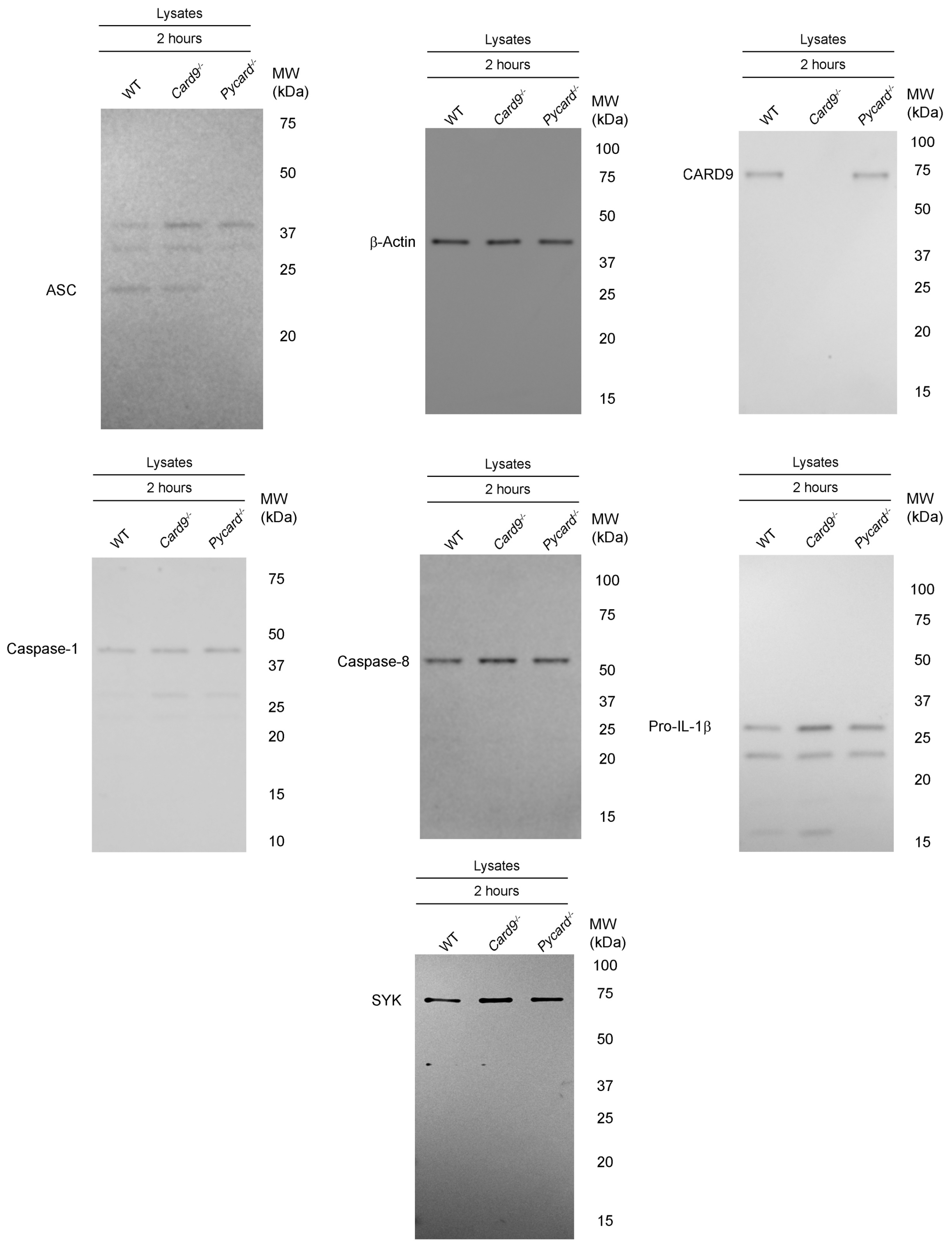

Supplementary Figure 12: Uncropped immunoblots presented in Figure 2-g, Lysates after 2 hours infection. Membranes were probed using the antibody indicated (ASC, β-Actin, CARD9, Caspase-1, Caspase-8, Pro-IL-1β and SYK).

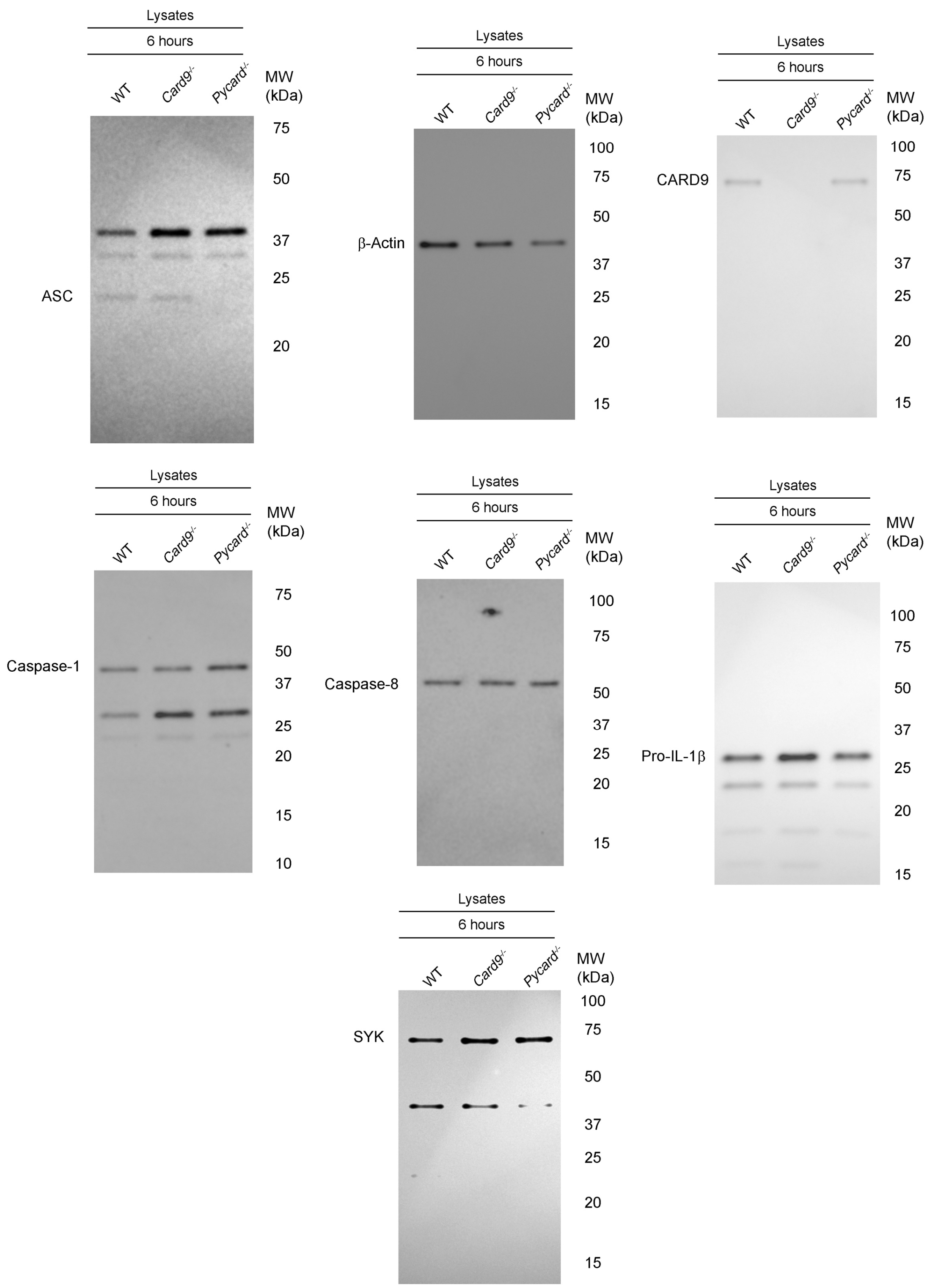

Supplementary Figure 13: Uncropped immunoblots presented in Figure 2-g, Lysates after 6 hours infection. Membranes were probed using the antibody indicated (ASC, β-Actin, CARD9, Caspase-1, Caspase-8, Pro-IL-1β and SYK).

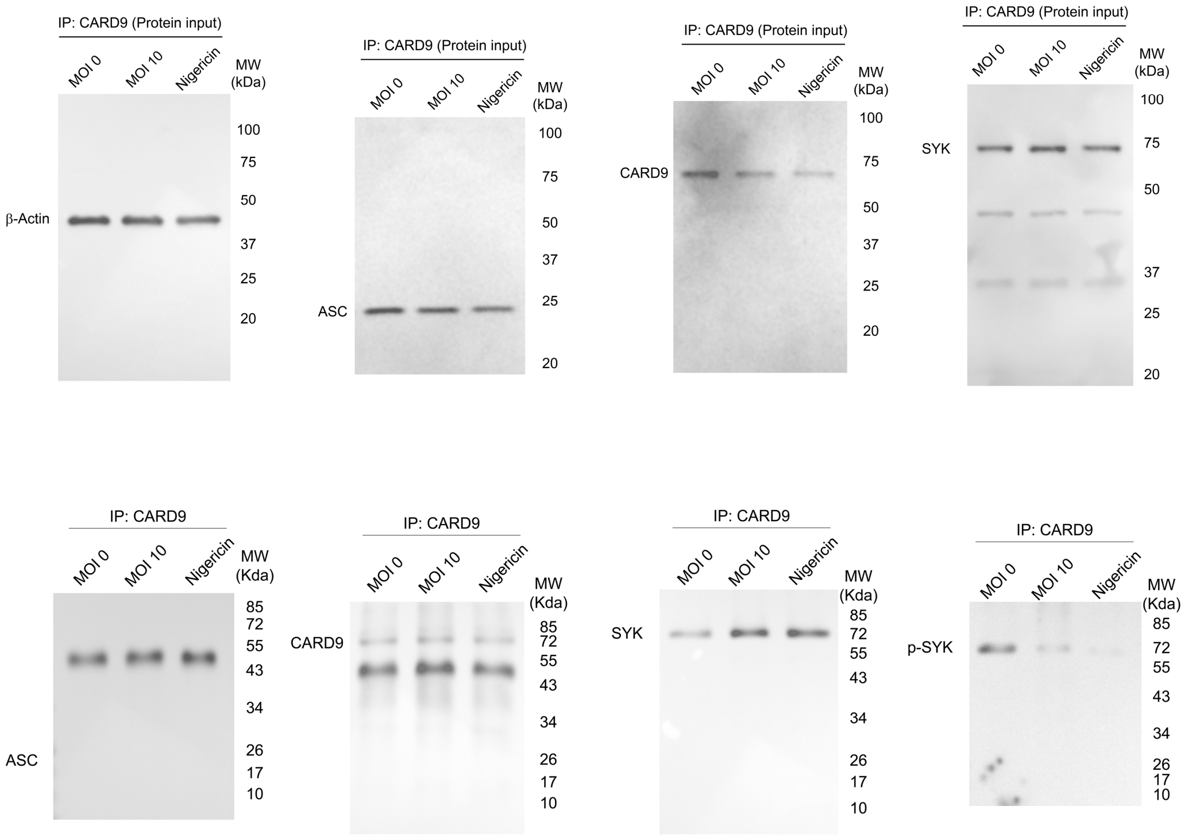

Supplementary Figure 14: Uncropped immunoblots presented in Figure 5-b, CARD9 co-IPs. Membranes were probed using the antibody indicated (β-Actin, ASC, CARD9, SYK and p-SYK).

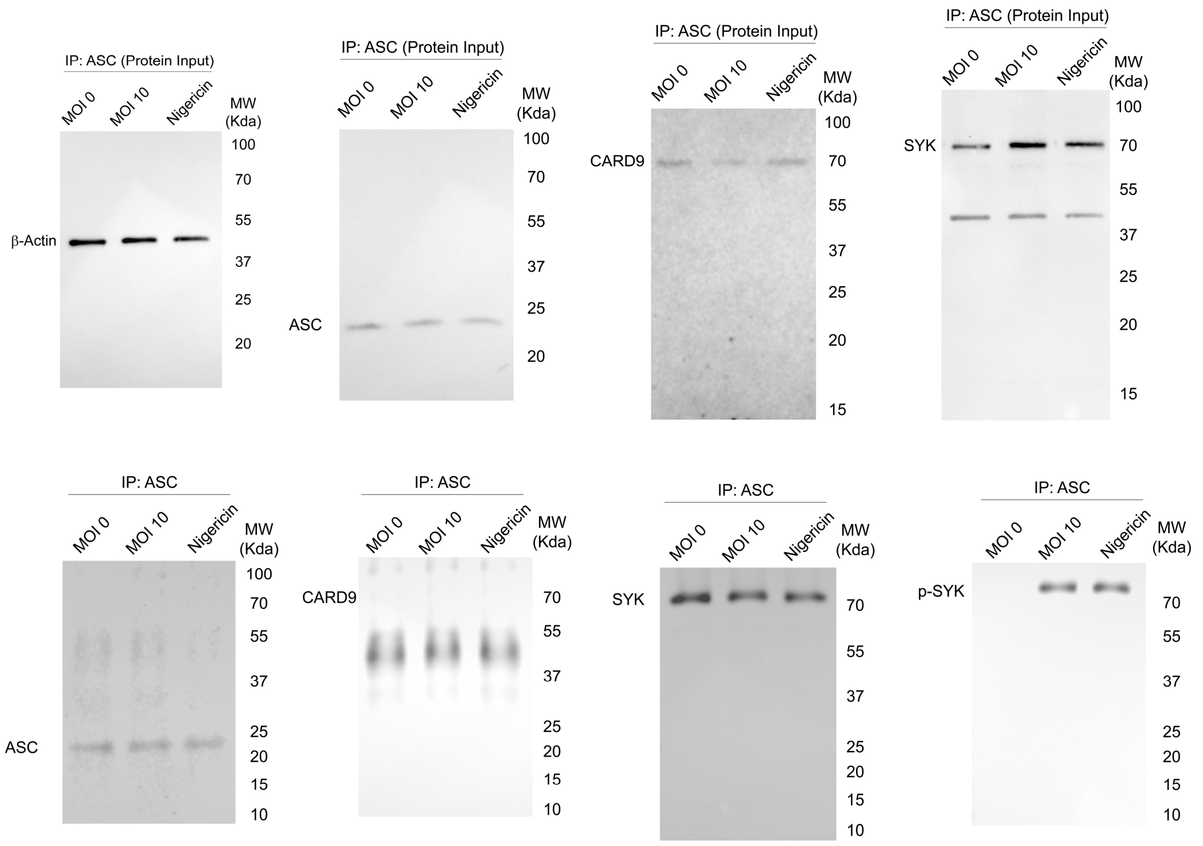

Supplementary Figure 15: Uncropped immunoblots presented in Figure 5-b, ASC co-IPs. Membranes were probed using the antibody indicated (β-Actin, ASC, CARD9, SYK and p-SYK).

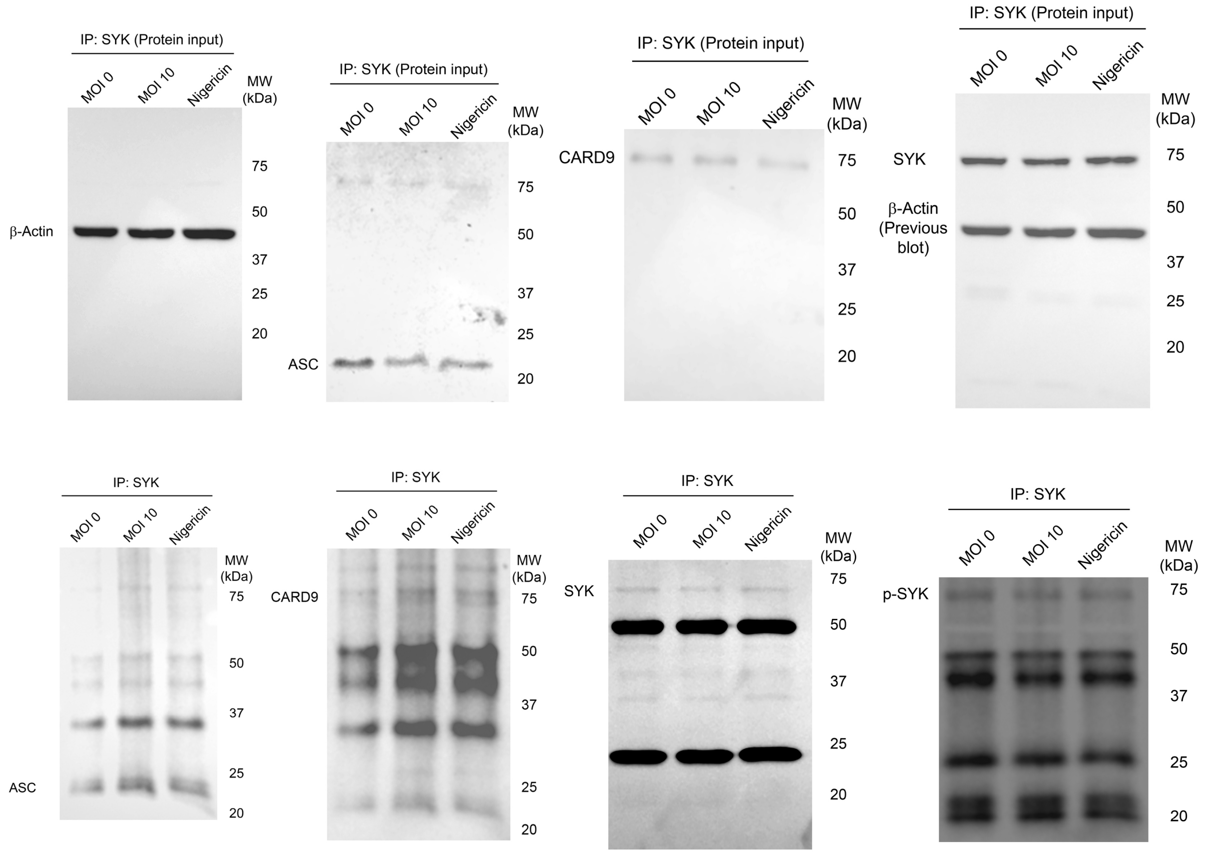

Supplementary Figure 16: Uncropped immunoblots presented in Figure 5-b, SYK co-IPs. Membranes were probed using the antibody indicated (β-Actin, ASC, CARD9, SYK and p-SYK).

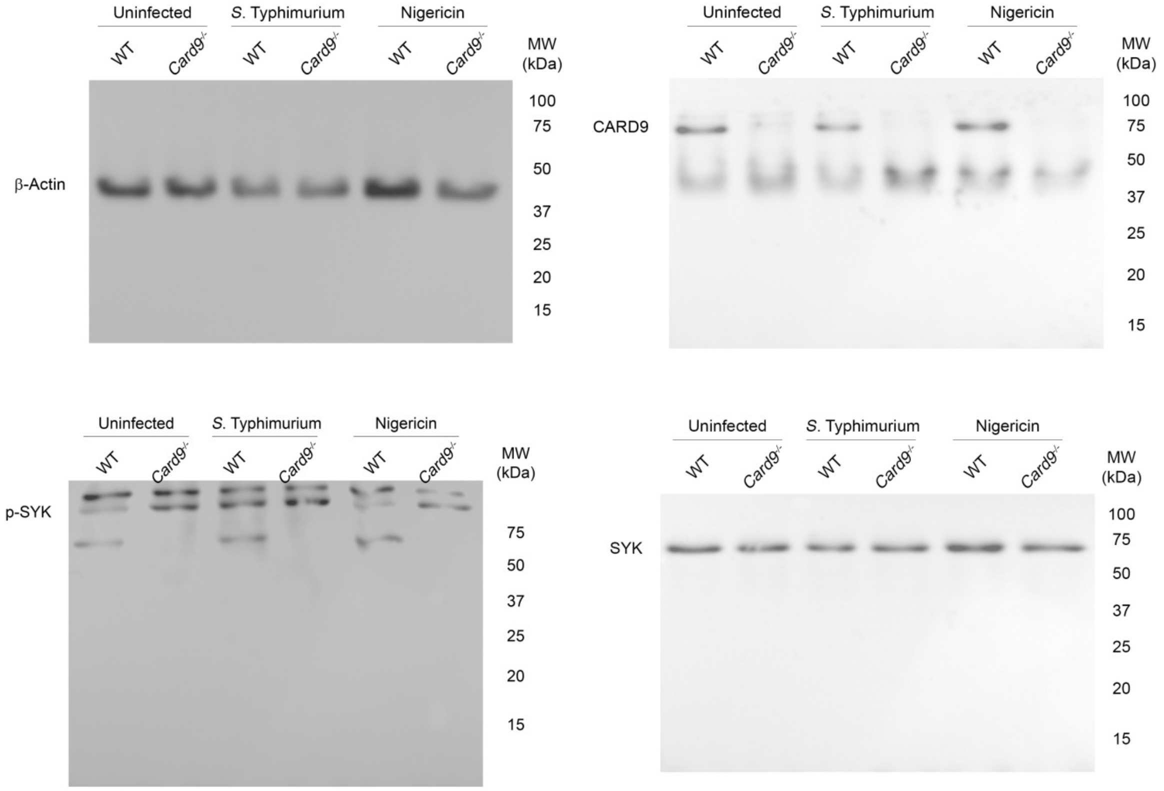

Supplementary Figure 17: Uncropped immunoblots presented in Figure 5-c. Membranes were probed using the antibody indicated (β-Actin, CARD9, SYK and p-SYK).

Fig. 8A

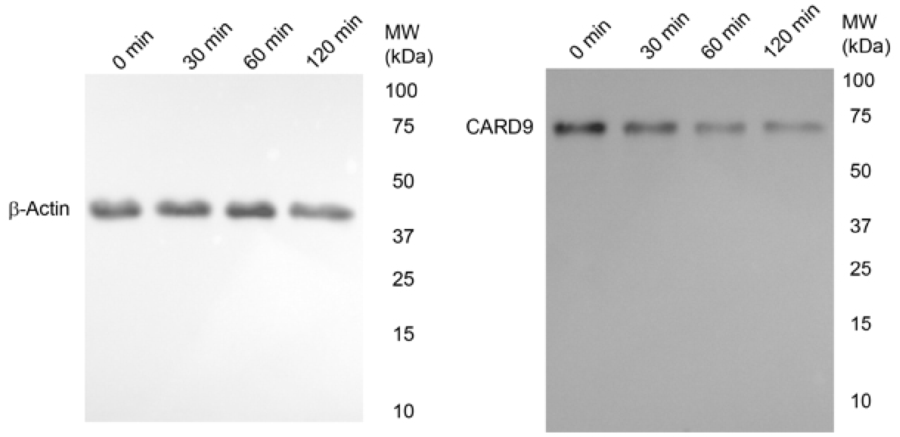

Fig. 8B

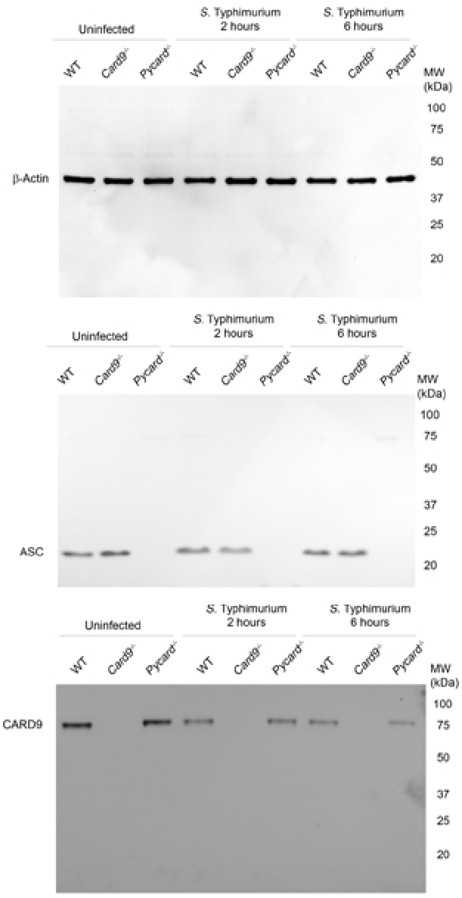

Fig. 8C

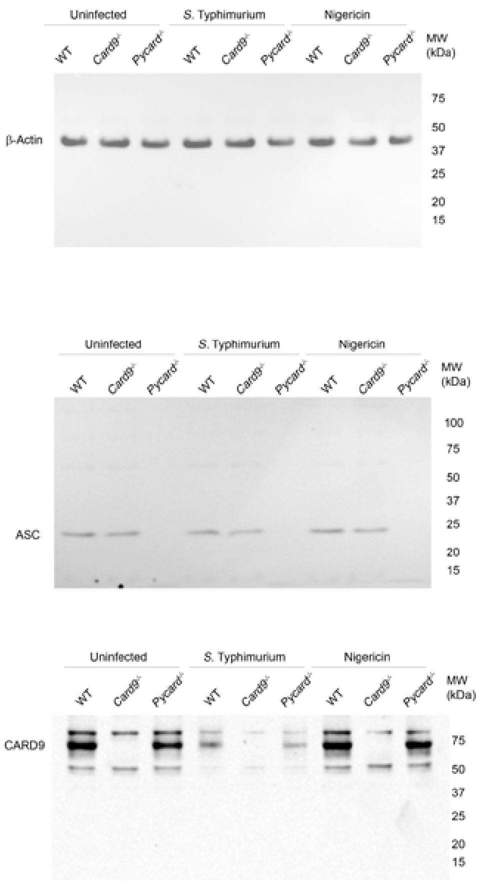

Supplementary Figure 18: Uncropped immunoblots presented in Figure 8. Membranes were probed using the antibody indicated (β-Actin, ASC, CARD9).

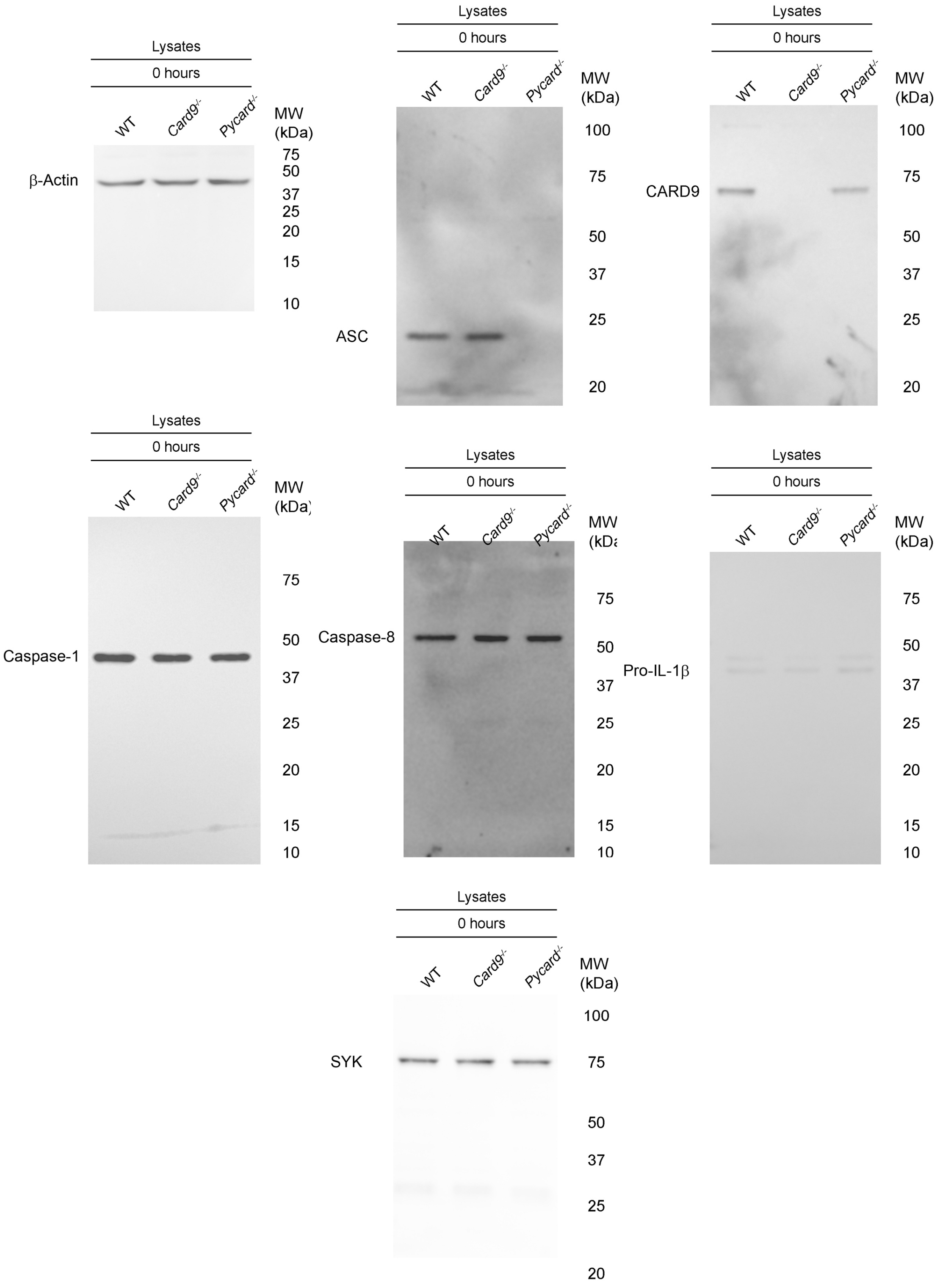

Supplementary Figure 19: Uncropped immunoblots presented in Supplementary Figure 2-j, Uninfected cell lysates. Membranes were probed using the antibody indicated (ASC, β-Actin, CARD9, Caspase-1, Caspase-8, Pro-IL-1β and SYK).

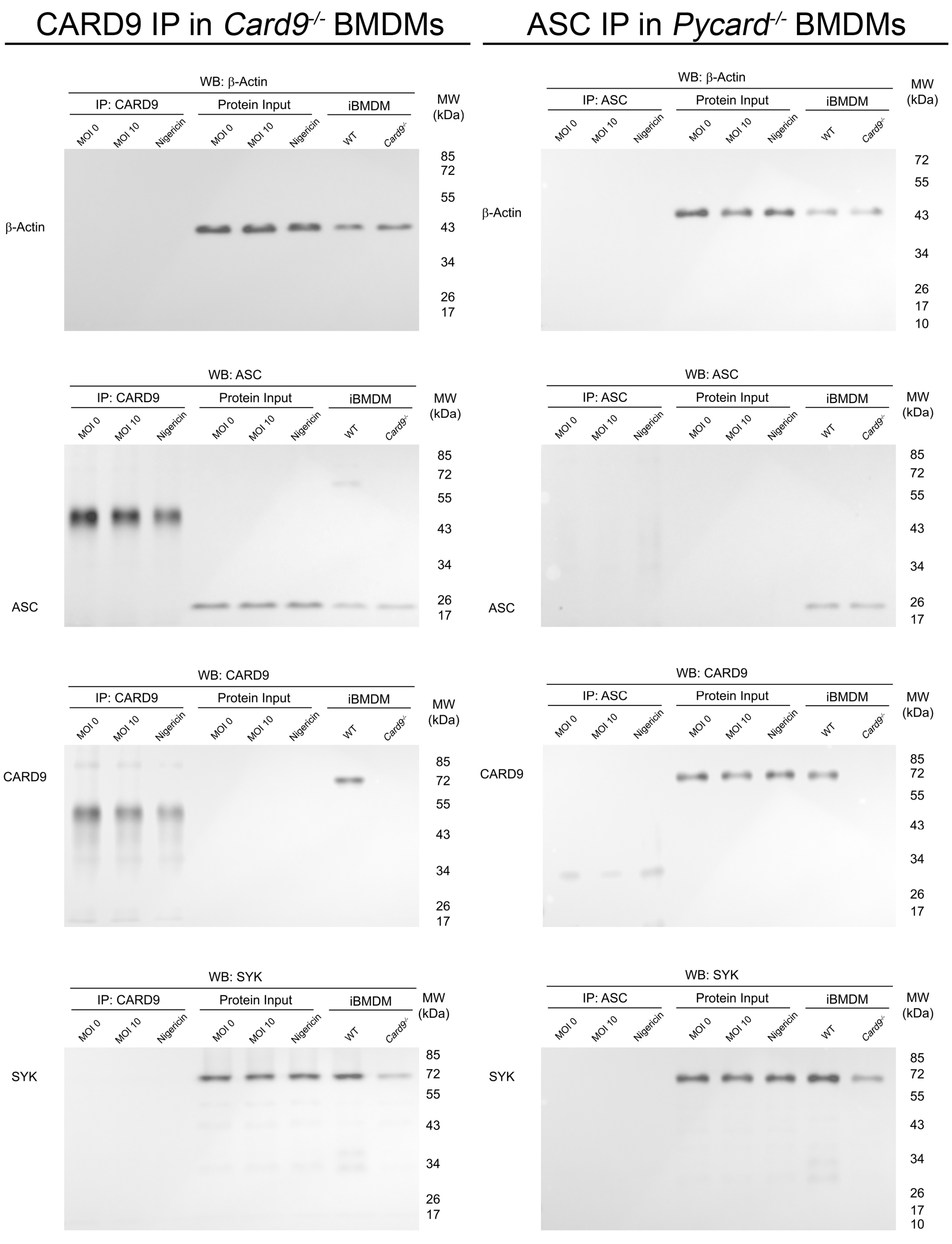

Supplementary Figure 20: Uncropped immunoblots presented in Supplementary Figure 4, IPs isotype control. Membranes were probed using the antibody (ASC, β-Actin, CARD9 and SYK). WT and *Card9*$^{-/-}$ iBMDMs cells were used as additional controls.

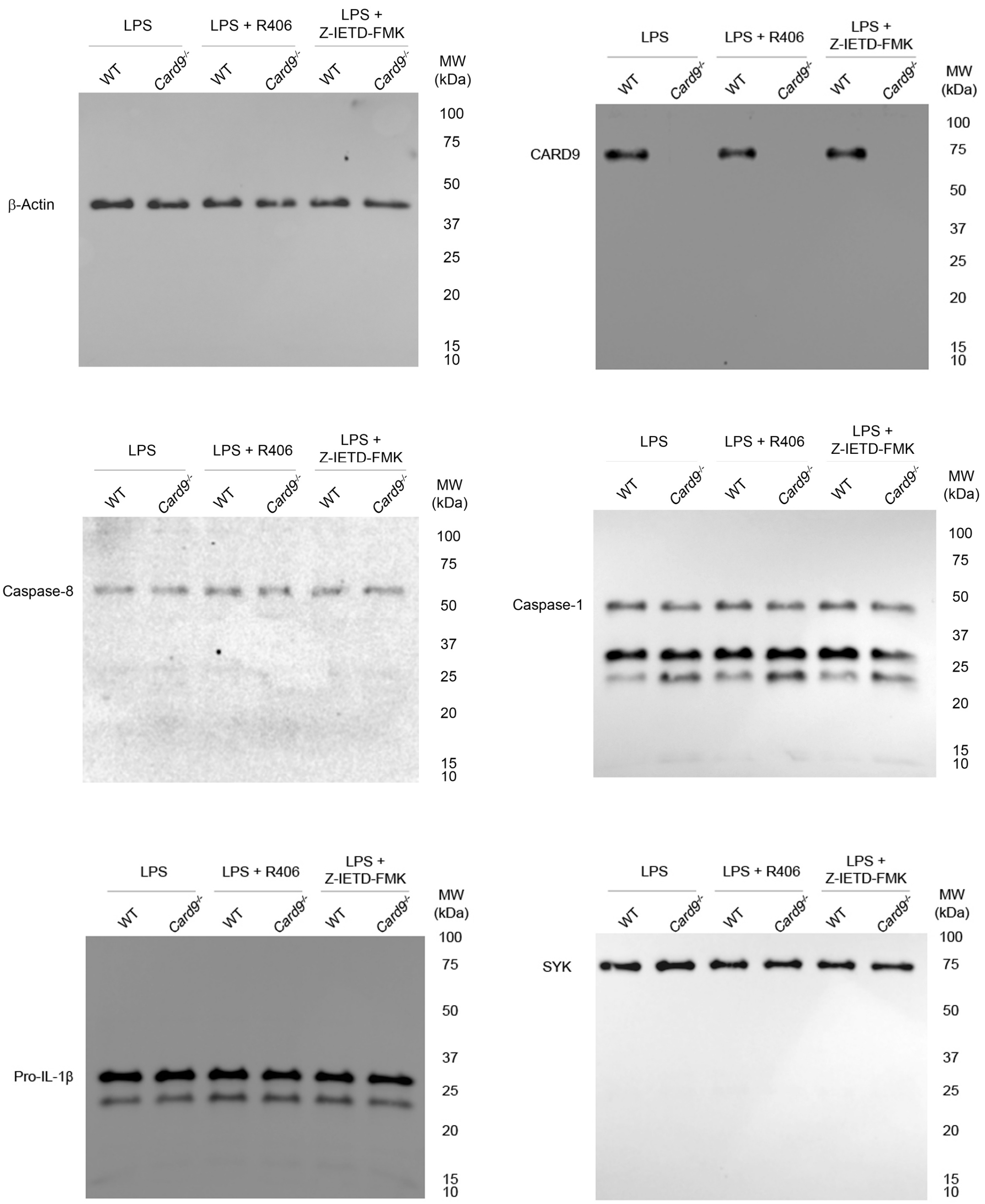

Supplementary Figure 21: Uncropped immunoblots presented in Supplementary Figure 5. Membranes were probed using the antibody indicated (β-Actin, CARD9, Caspase-1, Caspase-8, Pro-IL-1β and SYK).

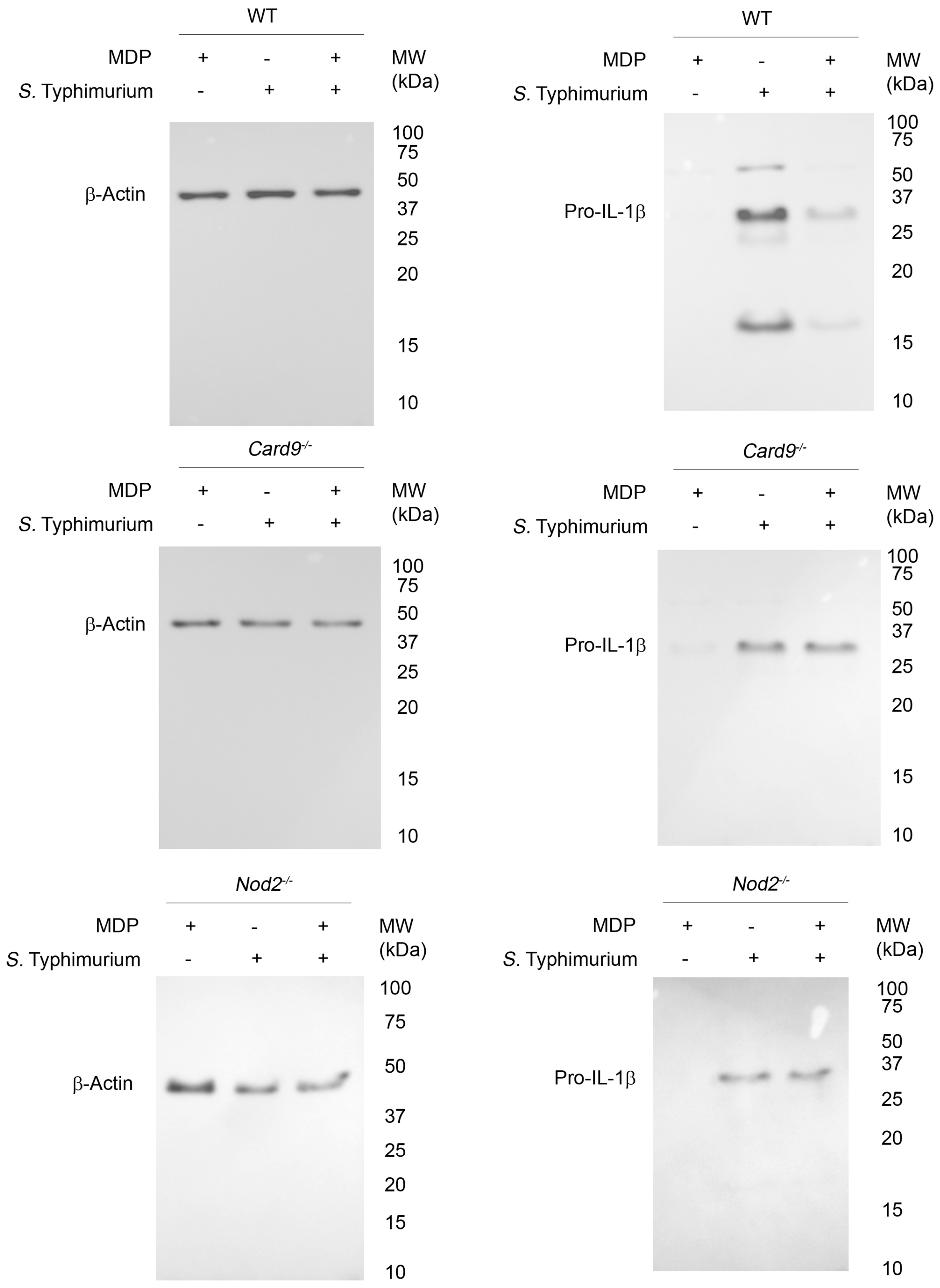

Supplementary Figure 22: Uncropped immunoblots presented in Supplementary Figure 7. Membranes were probed using the antibody indicated (β-Actin and Pro-IL-1β).

Supplementary Table 1: Pairs of primers used for the qPCR experiments.

Gene	Forward (5'-3')	Reverse (5'-3')	Amplicon size (bp)	Primer Bank ID
β-actin	GGCTGTATTCCCCTCCATCG	CCAGTTGGTAACAATGCCATGT	154	6671509a1
ASC	CTTGTCAGGGGATGAACTCAAAA	GCCATACGACTCCAGATAGTAGC	154	31560222a1
Bcl-10	CTTCAAGTAGAAAACGGGCTGG	GCACCTAGAGAGGTTGTTGGT	233	6753166a1
Caspase-1	ACAAGGCACGGGACCTATG	TCCCAGTCAGTCCTGGAAATG	237	6753282a1
Caspase-8	TGCTTGGACTACATCCCACAC	TGCAGTCTAGGAAGTTGACCA	169	33859520a1
FADD	GCGCCGACACGATCTACTG	TTACCCGCTCACTCAGACTTC	215	6753812a1
GAPDH	AGGTCGGTGTGAACGGATTTG	TGTAGACCATGTAGTTGAGGTCA	123	6679937a1
MALT-1	GGACAAAGTCGCCCTTTTGAT	TCCACAGCGTTACACATCTCA	165	27370250a1
NAIP5	TGCCAAACCTACAAGAGCTGA	CAAGCGTTTAGACTGGGGATG	203	5932014a1
NLRC4	TTGAAGGCGAGTCTGGCAAAG	GGCGCTTCTCAGGTGGATG	125	146198620c2
NLRP3	ATTACCCGCCCGAGAAAGG	TCGCAGCAAAGATCCACACAG	141	22003870a1
Pro-IL-18	GTGAACCCCAGACCAGACTG	CCTGGAACACGTTTCTGAAAGA	202	6680412c1
Pro-IL-1β	TTCAGGCAGGCAGTATCACTC	GAAGGTCCACGGGAAAGACAC	75	118130747c2
RANTES	GCTGCTTTGCCTACCTCTCC	TCGAGTGACAAACACGACTGC	104	7305461a1
SYK	CTACCTGCTACGCCAGAGC	GCCATTAAGTTCCCTCTCGATG	103	6755706a1
TNF-α	CCCTCACACTCAGATCATCTTCT	GCTACGACGTGGGCTACAG	61	7305585a1
